# The effects of ERN1 on gene expression during early rhizobial infection in *Lotus japonicus*

**DOI:** 10.3389/fpls.2022.995589

**Published:** 2023-01-17

**Authors:** Meng Liu, Hiromu Kameoka, Akiko Oda, Taro Maeda, Takashi Goto, Koji Yano, Takashi Soyano, Masayoshi Kawaguchi

**Affiliations:** ^1^ Division of Symbiotic Systems, National Institute for Basic Biology, Okazaki, Aichi, Japan; ^2^ Department of Basic Biology, School of Life Science, SOKENDAI (The Graduate University for Advanced Studies), Okazaki, Aichi, Japan

**Keywords:** LjERN1, *Lotus japonicus*, RNA sequencing, root nodule symbiosis, rhizobial infection

## Abstract

Legumes develop root nodules in association with compatible rhizobia to overcome nitrogen deficiency. Rhizobia enter the host legume, mainly through infection threads, and induce nodule primordium formation in the root cortex. Multiple transcription factors have been identified to be involved in the regulation of the establishment of root nodule symbiosis, including ERF Required for Nodulation1 (ERN1). ERN1 is involved in a transcription network with CYCLOPS and NODULE INCEPTION (NIN). Mutation of ERN1 often results in misshapen root hair tips, deficient infection thread formation, and immature root nodules. ERN1 directly activates the expression of *ENOD11* in *Medicago truncatula* to assist cell wall remodeling and *Epr3* in *Lotus japonicus* to distinguish rhizobial exopolysaccharide signals. However, aside from these two genes, it remains unclear which genes are regulated by LjERN1 or what role LjERN1 plays during root nodule symbiosis. Thus, we conducted RNA sequencing to compare the gene expression profiles of wild-type *L. japonicus* and *Ljern1-6* mutants. In total, 234 differentially expressed genes were identified as candidate LjERN1 target genes. These genes were found to be associated with cell wall remodeling, signal transduction, phytohormone metabolism, and transcription regulation, suggesting that LjERN1 is involved in multiple processes during the early stages of the establishment of root nodule symbiosis. Many of these candidate genes including *RINRK1* showed decreased expression levels in *Ljnin-2* mutants based on a search of a public database, suggesting that LjERN1 and LjNIN coordinately regulate gene expression. Our data extend the current understanding of the pleiotropic role of LjERN1 in root nodule symbiosis.

## Introduction

Legumes are able to overcome nitrogen deficiency by establishing root nodule symbiosis with nitrogen-fixing bacteria known as rhizobia. Inside the unique organs of symbiosis, root nodules, rhizobia convert atmospheric nitrogen into ammonium for plants’ benefits, in exchange for carbon source. Rhizobia enter legumes through cracks on the epidermis or, more commonly, through root hairs (Guinel and Geil, 2002). In *Lotus japonicus* and *Medicago truncatula—*two model legumes primarily used for studying root nodule symbiosis—the attachment of compatible rhizobia to the surface of a host root hair is observed to induce polar growth of the root hair tip, resulting in the formation of a “shepherd’s crook” structure. Rhizobia entrapped in the crook of the root hair multiply and form microcolonies. Subsequently, the rhizobia enter the root hair cell through the inward growth of tubular structures, which are called infection threads. The formation of infection threads is accompanied by the modification of the root hair cell wall, plasma membrane, and cytoskeletal structure. Concomitant with infection thread progression, cortical cells underneath the infection sites re-enter the cell cycle, divide, and form nodule primordia. Eventually, the infection threads reach the nodule primordia and release rhizobia into the nodule cells (reviewed by Oldroyd, 2013; Roy et al., 2019). One of the fundamental questions on the topic of root nodule symbiosis is how the legume–rhizobium association is established through root nodule symbiosis signal transduction.

The root nodule symbiosis signaling pathway has been observed to be initiated through the detection of rhizobia by the host legume. The host legume distinguishes Nod factors from compatible rhizobia *via* receptor kinases, including Nod Factor Receptor1 (LjNFR1)/LysM Receptor Kinase3 (MtLYK3), LjNFR5/Nod Factor Perception (MtNFP), and LjNFRe (Amor et al., 2003; Limpens et al., 2003; Madsen et al., 2003; Radutoiu et al., 2003; Murakami et al., 2018). These receptors transduce the signal to the nucleus and activate calcium signaling, which is then decoded by calcium and calmodulin-dependent protein kinase (LjCCaMK)/Doesn’t Make Infections 3 (MtDMI3) (Ehrhardt et al., 1996; Lévy et al., 2004; Miwa et al., 2006; Tirichine et al., 2006). Downstream of CCaMK activation, several transcription factors, including LjCYCLOPS/Interacting Protein of DMI3 (MtIPD3), Nodulation Signaling Pathway1 (NSP1), NSP2, NODULE INCEPTION (NIN), and ERF Required for Nodulation 1 (ERN1), form a network to reprogram gene transcription (Catoira et al., 2000; Kaló et al., 2005; Heckmann et al., 2006; Marsh et al., 2007; Messinese et al., 2007; Middleton et al., 2007; Cerri et al., 2016; Fonouni-Farde et al., 2016; Jin et al., 2016; Cerri et al., 2017; Murakami et al., 2006; Schauser et al., 1999; Smit et al., 2005; Yano et al., 2008; Singh et al., 2014; Yano et al., 2017). Among these transcription factors, NIN plays a central role in regulating the expression of genes associated with cell wall remodeling, cytoskeleton rearrangement, and cell division. Target genes of NIN encode proteins such as NODULATION PECTATE LYASE (LjNPL), an enzyme that mediates cell wall degradation during infection thread initiation (Xie et al., 2012; Liu et al., 2019a); SCAR-Nodulation (LjSCARN), a component of the actin regulatory complex that promotes the formation of new actin filaments in root hairs during infection thread development (Qiu et al., 2015); and Nuclear Factor-YA1 (LjNF-YA1/MtNF-YA1), a transcription factor that promotes cortical cell division for nodule organogenesis (Combier et al., 2006; Soyano et al., 2013). MtNF-YA1 also regulates infection thread formation *via* direct activation of *MtERN1* expression in *M. truncatula* (Laloum et al., 2014). Additionally, phytohormones are involved in root nodule symbiosis signaling (Buhian and Bensmihen, 2018; Lin et al., 2020). For example, auxin has been determined to positively affect infection thread formation (Nadzieja et al., 2018). Auxin can be detected in infected root hairs and dividing cortical cells (Suzaki et al., 2014). Mutation of *Auxin Response Factor 16a* (*MtARF16a*) leads to a reduced number of infection threads in *M. truncatula* (Breakspear et al., 2014). More recently it has been shown that IAA carboxyl methyltransferase 1 (IAMT1), which converts auxin (IAA) to its methyl ester (MeIAA), is required for nodule development and its metabolite MeIAA can induce *NIN* expression (Goto et al., 2022). Cytokinin is found to promote cortical cell division but represses infection in the epidermis. Exogenous application of cytokinin or gain-of-function mutations of the cytokinin receptor gene *LjLHK1* (*snf2 and snf5*) results in spontaneous root nodules, while *Ljlhk1* (*hit1*) mutants exhibit a reduced number of nodules and an increased number of infection threads (Murray et al., 2007; Tirichine et al., 2007; Heckmann et al., 2011; Miri et al., 2016; Liu et al., 2018). Another phytohormone, gibberellin (GA), has been identified to suppress root hair deformation and infection thread formation by degrading DELLA, a protein that interacts with CYCLOPS and NSP1-NSP2 to enhance symbiotic gene expression (Maekawa et al., 2009; Fonouni-Farde et al., 2016; Jin et al., 2016). In summary, root nodule symbiosis signaling involves various genes related to signal transduction, gene transcription regulation, and phytohormone metabolism.

Previously, we reported the function of LjERN1 in root nodule symbiosis signaling through the characterization of two allelic symbiotic mutant lines, *Ljern1-5* and *Ljern1-6*, which show deficiencies in their response to rhizobial infection. Like *Mtern1* mutants, *Ljern1* mutants display abnormal balloon-shaped root hair tips, a decreased number of infection threads, and immature root nodules (Cerri et al., 2017; Kawaharada et al., 2017a; Yano et al., 2017). Gain-of-function CCaMK or application of cytokinin does not induce spontaneous nodule production in *Ljern1* mutants (Kawaharada et al., 2017a). The corresponding gene ERN1 encodes an AP2/ERF transcription factor, which is expressed in infected root hairs and developing nodules (Middleton et al., 2007; Cerri et al., 2016; Cerri et al., 2017; Kawaharada et al., 2017a; Yano et al., 2017). The phenotype of *ern1* mutants and expression pattern of *ERN1* suggest that *ERN1* is needed for infection thread formation and promote nodule organogenesis. Two genes have been identified as targets of ERN1: *M. truncatula Early Nodulin11* (*MtENOD11*), which is involved in cell wall modification, and *L. japonicus Exopolysaccharide Receptor3* (*LjEpr3*), which is responsible for compatible rhizobial recognition (Andriankaja et al., 2007; Kawaharada et al., 2017b). To determine the role of ERN1 in *L. japonicus*, we conducted RNA sequencing (RNA-seq) to compare the gene expression profiles of wild-type (WT) plants and *Ljern1-6* mutant roots. *Ljern1-6* is a null allele mutant isolated from *L. japonicus* accession Miyakojima MG-20 and lacks approximately 10 kb including the entire length of *ERN1* (Yano et al., 2017). Although a transcriptome study has already been conducted in *Mtern1* mutants, the relationship between ERN1 and NIN differs between *M. truncatula* and *L. japonicus* and *L. japonicus* lacks an *ERN2* ortholog gene, suggesting that ERN1 may function differently between these two species (Andriankaja et al., 2007; Yano et al., 2017; Liu et al., 2019a; Liu et al., 2019b). In this present study, we found that of 3,763 genes induced by rhizobial infection in WT plants, 234 were significantly decreased in *Ljern1-6* mutants. These genes were found to be involved in processes including cell wall modification, signal transduction, phytohormone metabolism, and regulation of gene transcription. The differentially expressed genes (DEGs) with high fold change in *Ljern1-6* encoded expansins, pectin methylesterases (PMEs), and PME inhibitors (PMEIs), which are related to cell wall loosening and extensity. The *Ljern1* mutation was also found to reduce the expression of several LjNIN-targeting genes, which is consistent with our previous finding that LjERN1 and LjNIN coordinately affect downstream gene expression. This study extends our understanding of the regulatory network governed by LjERN1.

## Materials and methods

### Plant materials and growth conditions

*L. japonicus* accession Miyakojima MG-20 (Kawaguchi, 2000) was used as the WT. *Ljern1-6* mutant was generated from a MG-20 background by ion beam mutagenesis and carry approximately 10 kb deletion including the entire length of *ERN1* (Yano et al., 2017). *Ljnin-*9 was isolated from MG-20 by EMS mutagenesis (Suzaki et al., 2012). The mutant lines *Ljern1-1* and *Ljnin-2* were generated from a Gifu Background (Schauser et al., 1999; Kawaharada et al., 2017a). *L. japonicus* seeds were surface-sterilized in 10% NaClO and germinated in 1/2 B5 medium in a growth chamber at 24°C (16 hr light/8 hr dark). Four-day-old seedlings were transferred to vermiculite with B&D medium (Broughton and Dilworth, 1971). Two days after adaptation, seedlings were inoculated with *M. loti* MAFF303099. For the inoculation, 15 mL of *M. loti* liquid culture (OD600 = 1.8-2.0) was diluted in 1 L of B&D medium. Each cultivation pot containing 10 plants was poured twice with 50 mL of the medium.

### RNA sample preparation, library synthesis, qRT-PCR and sequencing

For each set of sampling conditions, three biological replicates were harvested (20 plants/sample) for total RNA isolation. Total RNA was isolated using the RNeasy Plant Mini Kit (QIAGEN). Genomic DNA was removed by treatment with DNase I (QIAGEN). The integrity of the RNA samples was determined using a bioanalyzer (Agilent). A 350 ng sample of RNA from each replicate was used for library preparation. Library construction was performed using the NEBNext^®^ Ultra™ II RNA Library Prep Kit for Illumina (NEB) and the NEBNext^®^ Poly(A) mRNA Magnetic Isolation Module (NEB). The concentration of the library was measured using a bioanalyzer (Agilent). RNA-seq was conducted on an Illumina HiSeq 2000 platform by single-end sequencing (read length = 50 bp).

For qRT-PCR validation, 50 ng of the total RNA from the extractions described above was used for each sample. The primer sequences were as follows: Ubiquitin_fwd, ACGGCTCTTATCAAGGGACCA; Ubiquitin_rev, CACTTGAGGTGGTTGTAGAGG; EXPB2_fwd, GGAGCTACGAAATGCTGGAA; EXPB2_rev, CACCATCCCCATCCTCATAC; Epr3_fwd, GTCTTCAGCGGGGTATTTGA; Epr3_rev, TGGCAGCAGTTTTGAACAAG; LOG4_fwd, CCTTGAAGAACTGTTGGAAATCATC; LOG4_rev, TCAAGCTTGCACATGAGGTCTTG; RINRK1(ALB1)_fwd, TATGCCTTTGGTGTGATGCT; RINRK1(ALB1)_rev, TCCACAGTCCATTCCTCTCT; NIN_fwd, AGCAAAGAGCATTGGTGTATGT; NIN_rev, AGCACCCTGCACTGAATCAA; Lj0g3v0070749_fwd, GGTTTGGAATTGGATGGTGTTG; Lj0g3v0070749_rev, AGGGACAAAATCAGAAGCACC; Lj0g3v0320499_fwd, GGTGCTGTTGATTTTATCTTTGGTG; Lj0g3v0320499_rev, GGTGCTGTTGATTTTATCTTTGGTG; Lj2g3v3339140_fwd, GGGAACGAACCCAAATGAAGAG; Lj2g3v3339140_rev, TCTCCTGTTACAAACTTGACCTTTG; Lj3g3v3751920_fwd, CAAGTGGTGGAGGATTGCTTTG; Lj3g3v3751920_rev, AGGTCAGCAACATCAAGACGT; Lj5g3v0642670_fwd, GGAGCTACGAAATGCTGGAA; Lj5g3v0642670_rev, CACCATCCCCATCCTCATAC.

### Data analysis

After sequencing, 66 bp of each sample was trimmed by trimmomatic (v0.33) and aligned to the *L. japonicus* genome assembly (v3.0) using Tophat2 (v2.1.0; Kim et al., 2013). Raw counts were calculated using HTSeq (v0.6.0; Anders et al., 2015) and analyzed using the edgeR package (v3.26.8; Robinson et al., 2010; McCarthy et al., 2012) in R (version 3.6.1; R Core Team, 2020; v1.1.453; R Studio Team, 2020). After filtering, genes that met our criteria (FC > 1.5 and FDR < 0.05) were defined as DEGs. For K-means clustering, the package factoextra was used to estimate the optimal number of clusters (Kassambara and Mundt 2020). A heat map was generated using Z-scores with the pheatmap package (Kolde, 2019). A Venn diagram was produced by BioVenn (Hulsen et al., 2008) and the gplots package (Warnes et al., 2022). BLAST and GO enrichment were conducted using BLAST^®^ command line applications (NCBI) and Blast2GO (Götz et al., 2008). FASTA files for BLAST were generated using the packages Biostrings (Pagès et al., 2020) and seqRFLP (Ding and Zhang, 2012). Protein kinase domains were predicted using SMART (Letunic et al., 2015; Letunic and Bork, 2018). The classification of transcription factors was based on PlantTFDB (Jin et al., 2014; Jin et al., 2015; Jin et al., 2017; Tian et al., 2020).The R packages openxlsx (Schauberger and Walker, 2020) and tidyverse (Wickham et al., 2019) were used to import and sort data.

### Plasmid construction and hairy root transformation

For promoter-GUS analysis, the modified binary vector, pCAMBIA1300 whose HPTII was replaced with GFP and *Asc*I site was introduced into the *Sma*I site was used (Kumagai and Kouchi, 2003). *RINRK1* promoter region (2,976 bp) was amplified using a primer set (5’-ATGGTACCCGCAATATGAGCCACTGCTA-3’, 5’-ATGGCGCGCCTTTTTGCTCTGTATTTTTTTGTTGAATTGTGAAGTTAG-3’). The promoter fragment was digested with *Kpn*I and *Asc*I, and ligated with the vector. *ALB1* terminator region (1,309 bp) was amplified using a primer set (5’-ATGGCGCGCCCCCAGAGTTTAGTTACCATGGAC-3’, 5’-ATGTCGACTGAACTTGCAGGAGGAGATG-3’). The terminator fragment was digested with *Asc*I and *Sal*I, and ligated with the vector. The reading frame cassette C.1 of the Gateway vector conversion system (Invitrogen) was inserted into *Asc*I site of the vector. GUSPlus gene in pCAMBIA1305.1 was amplified by 2 rounds PCR using 1^st^ primer set (5’-AAAAAGCAGGCTACCATGGTAGATCTGAGGGTAA-3’, 5’-AGAAAGCTGGGTTCACACGTGATGGTGATGGT-3’) and 2^nd^ primer set (5’-GGGGACAAGTTTGTACAAAAAAGCAGGCT-3’, 5’-GGGGACCACTTTGTACAAGAAAGCTGGGT-3’). The GUSPlus fragment was inserted into pDONR/ZEO (Invitrogen) *via* Gateway BP reaction (Invitrogen). The GUSPlus gene was transferred between the promoter region and the terminator region *via* Gateway LR reaction (Invitrogen).

For complementation analysis, the *RINRK1* (*ALB1*) cDNA amplified using a specific primer set (5’-AAGTCGACATGAGCCTAAAACCATTCTGGGC-3’, 5’-AAGCGGCCGCCATGTGTCAAATGATATGGATTTTTCATCT-3’) was inserted between SalI and NotI sites of pENTR-1A (Thermo Fisher Scientific), and subsequently transferred to pUb-GW-GFP (Maekawa et al., 2008) by the LR clonase reaction. *L. japonicus* hairy root transformation was basically performed with *Rhizobium rhizogenes* AR1193 as described previously (Díaz et al., 2005). Seedlings removed roots by cutting hypocotyls were co-cultured with *R. rhizogenes* harboring either *pUb-RINRK1-GFP* or its empty vector for four days, and then cultured on B5 ager plates for 13 days to generate hairy roots. Seedlings that formed hairy roots were inoculated with *M. loti* expressing DsRed (Maekawa et al., 2009) three days after transferring to sterile vermiculite. The number of nodules and infection threads formed in hairy roots displaying fluorescence from the GFP selection marker were counted at 22 dpi and 7 dpi, respectively, under an SZX16 stereomicroscope (Olympus).

## Results and discussion

### The LjERN1 mutation affected a number of rhizobial infection-induced genes

To gain a deeper understanding of the function of LjERN1 in the transcription network, we conducted an RNA-seq to compare gene expression in WT plants and *Ljern1-6* mutants, a strong allele of *Ljern1*, during the early stages of nodulation. *L. japonicus* wild type (WT; MG-20) and *Ljern1-6* were inoculated with *Mesorhizobium loti* MAFF 303099. Four time points were selected; samples from 0 day post rhizobial infection (dpi) were used as controls, while samples from 1, 2, and 3 dpi covered the period from root hair deformation initiation to infection threads and nodule primordia becoming visible. Three biological replicates, each consisting of 20 plants, were utilized for the sequencing. Reads were mapped to version 3.0 of the *L. japonicus* MG-20 genome. According to the *L. japonicus* Gifu genome recently released (Kamal et al., 2020), many gene IDs in MG-20 may correspond to the same gene in Gifu. We thus annotated corresponding Gifu gene IDs in **Table S1**.

**Figure 1:**
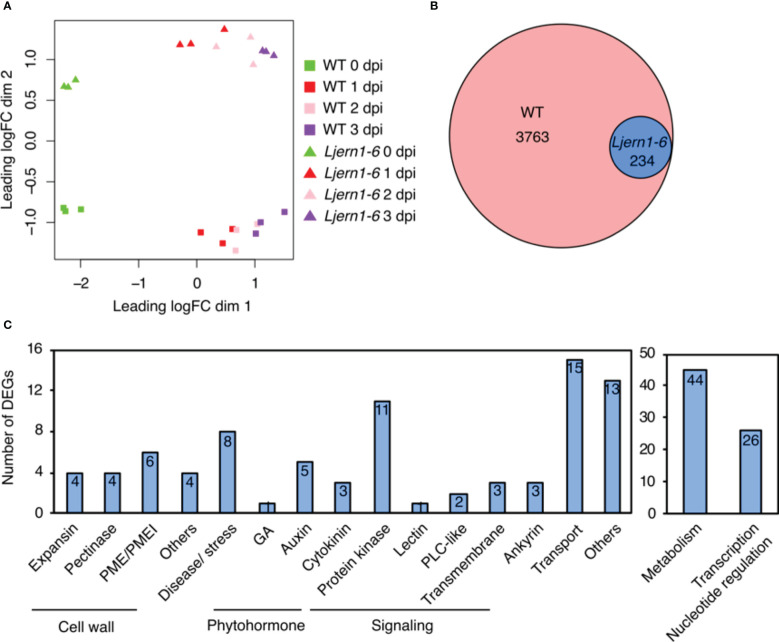
DEGs detected by RNA-seq in wild-type *L. japonicus* (MG-20) and *Ljern1-6* mutants. **(A)** Multidimensional scaling plot of the effect of rhizobial infection on WT plants and *Ljern1-6* mutants. **(B)** Proportional Venn diagram (Hulsen et al., 2008) shows the number of rhizobial infection-induced genes in WT and DEGs with decreased expression in *Ljern1-6* mutants. **(C)** Classification of DEGs with decreased expression in *Ljern1-6* mutants.

A multidimensional scaling plot demonstrated the separation of the WT and *Ljern1-6* samples collected at 0 and 1−3 dpi (**Figure 1A**). After quality control and filtering, 47,232 genes were detected by RNA-seq. The expression of 3763 of these genes was up-regulated in response to rhizobial infection [fold change (FC) > 1.5, false discovery rate (FDR) < 0.05; **Table S1**]. We then subsequently grouped these 3,763 infection-induced DEGs into 7 clusters based on a K-means method (**Figure S1**). Genes in Cluster 2 and 7 showed lower transcript levels in *Ljern1-6* than in the WT. To achieve better separation of genes affected by the *LjERN1* mutation, we selected genes with higher fold change in *Ljern1-6* compared with the WT (FC > 1.5) from Cluster 2 and 7 for further analyses. In total, 234 genes were identified as DEGs, with decreased expression in *Ljern1-6* (**Figure 1B**; **Table S2**). To verify the RNA-seq result, we selected a few genes that were observed to change in expression by a large amount and checked their expression levels using qRT-PCR; *NIN* and *LjEpr3* were used as positive controls. The fold changes indicated by qRT-PCR were comparable to those indicated by RNA-seq (**Figure S2A**). We also selected several known symbiosis genes from a recent review by Roy et al. (2019) and further examined their expression levels in the WT and *Ljern1-6* plants (**Figure S2B**). The expression of genes that are essential for root nodule symbiosis, such as *LjCCaMK*, *LjNSP1*, and *LjCYCLOPS*, were increased in the WT, further confirming the result of RNA-seq. Notably, a few symbiosis genes have exhibited reduced expression levels in the *Ljern1-6* mutants, including *LjNPL*, *LONELY GUY4* (*LjLOG4*), *Cytokinin oxidase/dehydrogenase3* (*LjCKX3*), *LjCHIT5*, *LjNIN*, *LjNF-YA1*, *LjASL18*, *LjNOOT*, *LjCBS1*, and *LjRPG*.

Gene Ontology (GO) analysis revealed that DEGs with decreased expression in *Ljern1-6* were enriched in a variety of functions, including cell wall modification, signal transduction, transcription, and response to phytohormones (**Table S3**). Based on this result and BLASTP hits in *M. truncatula* and *Arabidopsis thaliana*, we manually classified the functions of 234 DEGs with decreased expression in *Ljern1-6* into 17 categories (**Figure 1C**; **Table S4**; genes without any annotation were removed).

### DEGs with decreased expression and high fold change in *Ljern1-6* mutants were associated with cell wall modification

**Figure 2:**
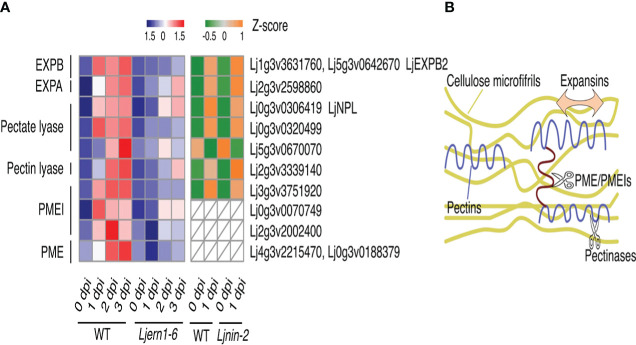
Cell wall-associated DEGs in WT plants and mutants. **(A)** Heatmap showing the expression levels of cell wall-associated DEGs in the WT and mutants at different time points. Left grid, Z-scores of genes in the WT (MG-20) and *Ljern1-6* mutants at 0, 1, 2, and 3 dpi; right grid, Z-scores of genes in the WT (Gifu) and *Ljnin-2* mutants at 0 and 1 dpi. **(B)** Illustration of the role of expansins, PEMs, PMEIs, and pectinases in the cell wall modification process.

Root hair curling and subsequent infection thread formation require the synthesis and degradation of the cell wall. Nodule primordium development also necessitates the synthesis of new cell walls (Brewin, 2004; Gage, 2004; Lohar et al., 2006). Transcriptome analyses identified cell wall-associated genes encoding expansin, peroxidases, and proteases that were induced by rhizobial infection in both *M. truncatula* and *Glycine max* (Breakspear et al., 2014). We found that LjERN1 mutation affected the expression of 13 genes encoding expansins, pectinase (pectate and pectin lyase), PMEs, and PMEIs post rhizobial infection (**Figure 2A**), suggesting that LjERN1 may be involved in regulating cell wall-associated processes.

Among these cell wall-related genes, the expression level of *β-expansin2* (*LjEXPB2*) was highly decreased in *Ljern1-6* mutants (**Figure 2A**; **Figure S3**). Expansins loosen the cellulose microfibrils by disintegrating the polysaccharide network, causing cell wall creep during cell growth (**Figure 2B**; Majda and Robert, 2018; Mohanty et al., 2018). Three expansin-encoding genes were induced by rhizobial infection in the WT, and *LjEXPB2* expression was the most affected in *Ljern1-6* mutants, especially at 1 dpi. *LjEXPB2* may be involved in promoting root hair growth and infection thread formation through loosening of the cell wall at an early infection stage. It has been speculated that expansin plays a role in root nodule symbiosis. Previously, increased expression of *EXP1* was detected in infected roots and nodules in *Melilotus albus* (Giordano and Hirsch, 2004). Immunoblotting showed that in *Pisum sativum* EXP1 is localized to infection thread walls (Sujkowska et al., 2006). Additionally, overexpression of *EXPB2* in *G. max* increased the number of root hairs, infection threads, and root nodules (Li et al., 2015).

Pectins are embedded in the cellulose microfibrils of cell walls, which enhance cell wall strength (Majda and Robert, 2018; **Figure 2B**). The degradation of cell walls is necessary for the continuous growth of infection threads therefore involves the removal of pectins (Allan Downie and Xie, 2015). *LjNPL* encodes a pectate lyase and is required for rhizobia to penetrate root hair cell walls. In *Ljnpl* mutants, rhizobia are entrapped in the root hair tip and cannot develop into infection threads (Xie et al., 2012). The expression of one pectin lyase gene, two pectate lyase genes, and *LjNPL* was decreased in *Ljern1-6* mutants (**Figure 2A**); Suppression of these genes may interrupt cell wall degradation in *Ljern1-6* mutants.

The *Ljern1* mutation also strongly affected genes encoding two cell wall-associated enzymes, PMEs, and their inhibitors, PMEIs (**Figure 2A**). PMEs have been identified to modify the crosslinks among different pectin domains through demethylesterification, thus softening the cell wall (Majda and Robert, 2018). This activity is negatively regulated by PMEIs (**Figure 2B**). Among the six rhizobial infection-induced PME- and PMEI-encoding genes, *Lj3g3v3751920* and *Lj0g3v0070749* showed the highest fold change in *Ljern1-6* mutants at 1 dpi, suggesting that PMEI may function during an early stage of infection. In *A. thaliana*, AtPMEI2 interacts with AtPME1 to regulate cell wall stability at the apex of the pollen tube. Transient expression of *AtPMEI2* was observed to increase the pollen tube length in tobacco, suggesting a role of PMEI in promoting polar cell growth (Röckel et al., 2008). In *M. truncatula*, a PME gene, *MtPER*, was proposed to have been recruited from the pollen tube elongation process to root nodule symbiosis (Rodríguez-Llorente et al., 2004), which suggests that PME and PMEIs may share similar functions in pollen tube growth and infection thread formation. The reduced expression of *LjPMEIs* may disrupt the cell wall extensity of the *Ljern1-6* root hair and lead to abnormal tip growth.

Because the expression of *LjNIN* was decreased in *Ljern1-6* mutants (**Figure S2**; Liu et al., 2019b), we compared the transcription profile of *Ljern1-6* obtained from the present RNA-seq analysis with DNA array data of the WT (Gifu) and *Ljnin-2* from the *Lotus japonicus* Gene Expression Atlas (Lotus Base; Mun et al., 2016) to determine whether the reduced expression of these cell wall-related genes was a secondary effect of *LjNIN*. Of all DEGs with decreased expression in *Ljern1-6* mutants, corresponding probes of 128 were detected in the DNA array. The expression of 32 genes was *LjNIN*-dependent (FC of wild type/nin > 1.5 at 1 dpi) (**Table S5**), while the remaining 32 genes were *LjNIN*-independent (**Table S6**). The expression of pectinase-, PME- and PMEI-encoding genes was reduced in *Ljnin-2* mutants, whereas the expansin genes showed a comparable expression level in the wild type (Gifu) (**Figure 2A**). The regulation of *LjEXPB2* may be mainly dependent on LjERN1, making *LjEXPB2* a candidate LjERN1 target. We examined *LjEXPB2* expression in MG-20-derived *Ljern1-6* and *Ljnin-9* roots using qRT-PCR and found that the induction of *LjEXPB2* expression in response to rhizobial infection appeared to be LjERN1-dependent and LjNIN-independent (**Figure S3**). Cell wall-associated genes such as *LjEXPB2* may contribute to cell wall loosening, degradation, and reconstruction during infection thread formation and nodule development. The reduced expression of *LjEXPB2* could interrupt cell wall dynamics in *Ljern1-6* mutants.

### Expression of 9 protein kinase genes was decreased in *Ljern1-6* mutants

**Figure 3:**
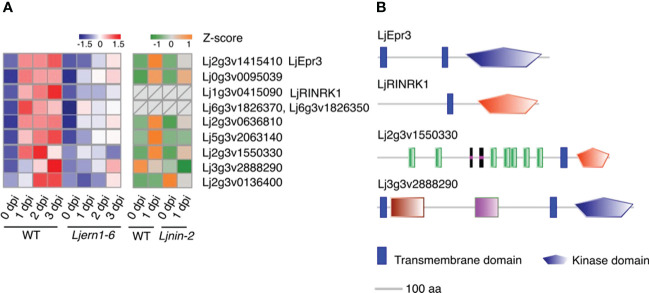
Expression of differentially expressed kinase genes in WT and mutants. **(A)** Heatmap of changes in the expression of differentially expressed kinase genes in the WT (MG-20) and *Ljern1-6* mutants (left grid) and WT (Gifu) and *Ljnin-2* mutants (right grid). **(B)** Receptor-like kinase prediction. Extracellular, transmembrane, and kinase domains were predicted using SMART (Letunic et al., 2015; Letunic and Bork, 2018); *LjEpr3* and *LjRINRK1* were used as positive controls. Blue stands for S_TKc, Serine/Threonine protein kinases, catalytic domain while red stands for STYKc, protein kinase; unclassified specificity.

**Figure 4:**
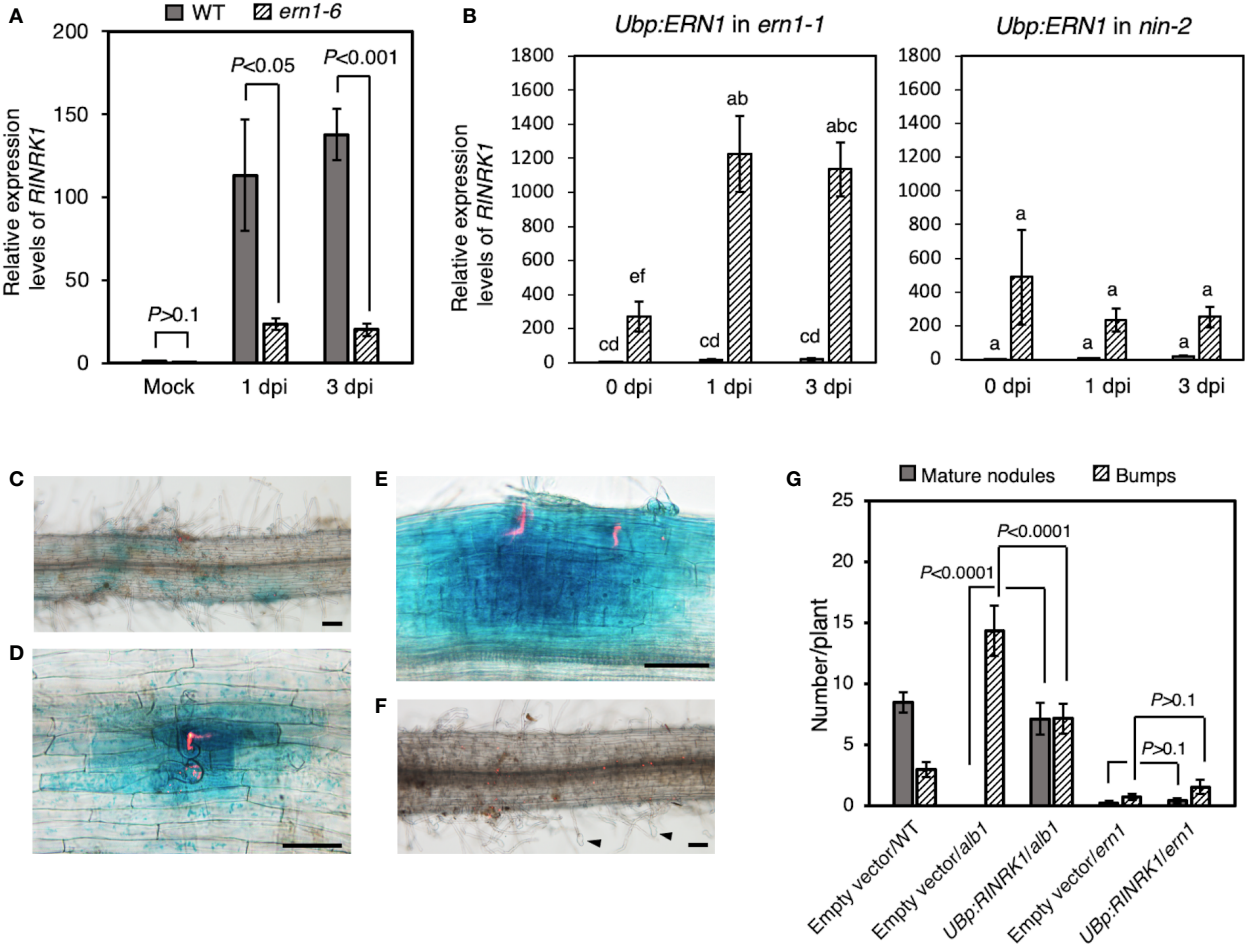
Positive regulation of *RINRK1* by ERN1. **(A)** Relative expression levels of *RINRK1* in WT and *Ljern1-6.* **(B)** Relative expression levels of *RINRK1 in Ljern1-1 and Ljnin-2.* Roots transformed with an empty vector (gray bar) or *UBp : ERN1* (stripe bar). Data are means of fold changes normalized to *Ubiquitin* and displayed relative to the empty vector control of *Ljern1-1* at 0 dpi (non-inoculation). Error bars indicate SE (n = 3, sample size = 12 plants). Statistical analysis was performed by ANOVA followed by Tukey's HSD test (P<0.05). GUS expression from *RINRK* promoter after inoculation with DsRed-labeled *M. loti*. Roots of WT **(C–E)** or the *Ljern1-6* mutants **(F)**. Scale bars, 200μm. Arrow heads: balloon-shaped root hairs. **(G)** Effects of overexpression of *RINRK1* on nodulation in the hairy roots of the *alb1* and *Ljern1-1* mutants.

Nine protein kinase genes showed decreased expression in *Ljern1-6* mutants, including two well-studied receptor-like kinase (RLK) genes, *LjEpr3* and *LjRINRK1* (**Figure 3A**; **Figure S3**). LjEPR3 recognizes rhizobial exopolysaccharides and regulates rhizobial passage through the host epidermal cell layer (Kawaharada et al., 2015; Kawaharada et al., 2017b). LjRINRK1 is likely involved in positive feedback with *LjNIN* and amplifies the infection signal (Li et al., 2019). In order to confirm whether ERN1 up-regulates the expression of *RINRK1*, the transcript accumulation of *RINRK1* was compared by qRT-PCR in the background of MG-20 and *Ljern1-6*. In MG-20, *RINRK1* was significantly induced 1 dpi, and its expression was further increased 3 dpi. On the other hand, the induction decreased at about 1/5 to 1/7 of MG-20 in the *Ljern1-6* background (**Figure 4A**). In addition, constitutive expression of *ERN1* tended to increase the expression of *RINRK1* in *Ljnin-2* mutants, under both uninfected and infected roots (**Figure 4B**). On the other hand, *UBp : NIN* also induced *RINRK1* expression in uninfected and infected roots of *Ljern1-1* (**Figure S4**). These results suggest that transcriptional activation of *RINRK1* is NIN- and ERN1-dependent. Indeed, *UBp : ERN1* failed to fully restore *RINRK1* expression in the *nin-2* mutant, suggesting that both NIN and ERN1 are required for full *RINRK1* expression.

Then, to analyze the spatial expression pattern of *RINRK1*, we constructed a GUS reporter carrying its promoter (2,976 bp), and transformed to the hairy roots *via R. rhizogenes*. Infection threads are visualized when infected with DsRED-labeled rhizobia. *RINRK1* was expressed in the region where the infection threads were formed (**Figure 4C**), and then strongly expressed in the divided cortical cells and nodule primordia (**Figures 4D, E**). On the other hand, in the *Ljern1-6* background, no expression of *GUS* was observed even in the region where the characteristic root hair deformation was observed after the infection (**Figure 4F**). Subsequently, we investigated whether the failure of the infectious process in *Ljern1-6* could be suppressed by constitutive expression of *RINRK1*. Prior to the experiment, we confirmed that the efficiency of formation of mature nodules was increased by introducing *RINRK1* into the *alb1* mutant carrying a mutation in *RINRK1* (**Figure 4G**). When *RINRK1* was overexpressed in the hairy roots of the *Ljern1-6* mutant, nodule primordia and mature nodules tended to increase slightly compared to the lines in which the empty vector was introduced (**Figure 4G**). On the other hand, the average numbers of ITs (± SD) was 1.40 ± 1.62 (n = 15) in the *Ljern1-1* hairy roots transfected with the empty vector, and 1.47 ± 2.06 (n = 15) in the hairy roots transfected with the *UBp : RINRK1*. Phenotypic suppression was not sufficient, suggesting that host factors other than RINRK1 regulate downstream of ERN1 also contributed significantly to the infection process.

In addition to these two kinases, the SMART analysis has revealed that the predicted proteins of Lj2g3v1550330 and Lj3g3v2888290 feature RLK structures (extracellular domain + transmembrane domain + kinase domain; **Figure 3B**; Shiu and Bleecker, 2001; Letunic et al., 2015; Letunic and Bork, 2018). Lj0g3v0095039 did not contain typical RLK domains, but its best BLASTP hit in *A. thaliana* was predicted to be RLKs (Shiu and Bleecker, 2001). The prediction that these three DEGs encode RLKs implies that they play a role in signal transduction. The reduced expression of these kinase genes may inhibit the ability of *Ljern1-6* mutants to identify compatible rhizobia and transduce signals to the nucleus. Similarly, in *Mtern1* mutants, the expression of 30 kinase genes was reduced (Liu et al., 2019a), suggesting that ERN1 may have similar functions in signal transduction in two species.

### *LjERN1* mutation affected the expression of four phytohormone-related genes

**Figure 5:**
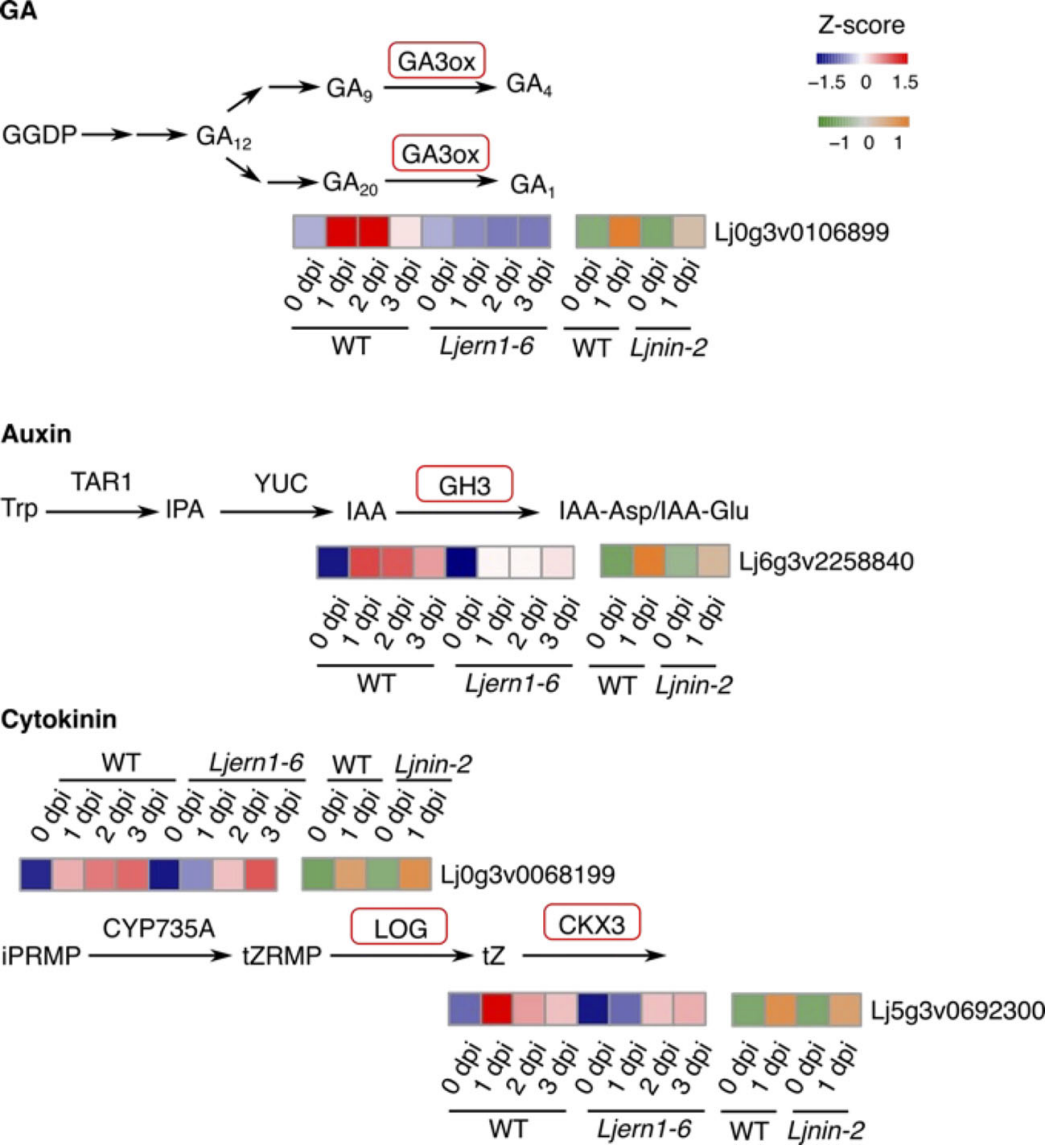
Phytohormone-associated DEGs in WT and mutants. Simplified GA, auxin, and cytokinin biosynthesis pathways were shown. Genes encoding enzymes with red frames were significantly reduced in *Ljern1-6* mutants. Heatmaps show the expression patterns of corresponding genes in WT and mutants.

Previous studies have shown that *Ljern1* alleles are deficient in response to cytokinin and auxin signaling during root nodule symbiosis (Kawaharada et al., 2017a; Nadzieja et al., 2018), which implies a role of *LjERN1* in phytohormone signaling. In the present *Ljern1-6* dataset, we found that four genes associated with phytohormones showed decreased expression levels (**Figure 5**). *GIBBERELLIN 3 BETA-HYDROXYLASE1* (*LjGA3ox1*) encodes an enzyme that converts GA to a bioactive form. GA negatively regulates root nodule symbiosis through the degradation of DELLA proteins (Fonouni-Farde et al., 2016; Jin et al., 2016). The decreased expression of *LjGA3ox1* in *Ljern1-6* mutants may be a secondary effect of decreased numbers of infection threads in these mutants.

Nadzieja et al. (2018) showed that auxin is necessary for infection thread formation in *L. japonicus*. The expression of an auxin-related genes, *Gretchen Hagen3* (*LjGH3*), was reduced in *Ljern1-6* mutants in the present study. GH3 balances auxin levels by catalyzing the conjugation of auxin. It is possible that *LjERN1* is related to auxin homeostasis, which, in turn, affects root nodule symbiosis.

Consistent with a previous report (Reid et al., 2017), we found that *LjLOG4*, which encodes an enzyme catalyzing the conversion of cytokinin precursors to a bioactive form, showed decreased expression levels in *Ljern1-6* mutants (**Figure S3**). Subsequently, the expression level of *LjCKX3*, which was involved in the breakdown of cytokinin, was also decreased (**Figure 5**). Cytokinin has been identified to suppress infection thread formation but promotes cortical cell division (Murray et al., 2007; Tirichine et al., 2007; Miri et al., 2016). Although *LjERN1* is not essential for nodule organogenesis as all *Ljern1* allele lines are able to produce root nodules, *LjERN1* mediates the formation of cytokinin-induced spontaneous root nodules (Kawaharada et al., 2017a). The decreased expression of *LjLOG4* and *LjCKX3* may explain the lack of spontaneous root nodules in *Ljern1* lines.

The expression levels of all four phytohormone-related DEGs were increased in *Ljern1-6* mutants to levels comparable with those in the wild type after 2 dpi, suggesting that LjERN1 may be involved in their regulation at an early stage. *LjGA3ox1* and *LjGH3* have displayed decreased expression levels in *Ljnin-2* lines, suggesting that their regulation may be dependent on both LjERN1 and LjNIN. LjERN1 may be more involved in the regulation of the cytokinin pathway, since *LjLOG4* and *LjCKX3* expression was decreased in *Ljern1-6* mutants but not in *Ljnin-2* mutants.

### LjERN1 and the transcription network

**Figure 6:**
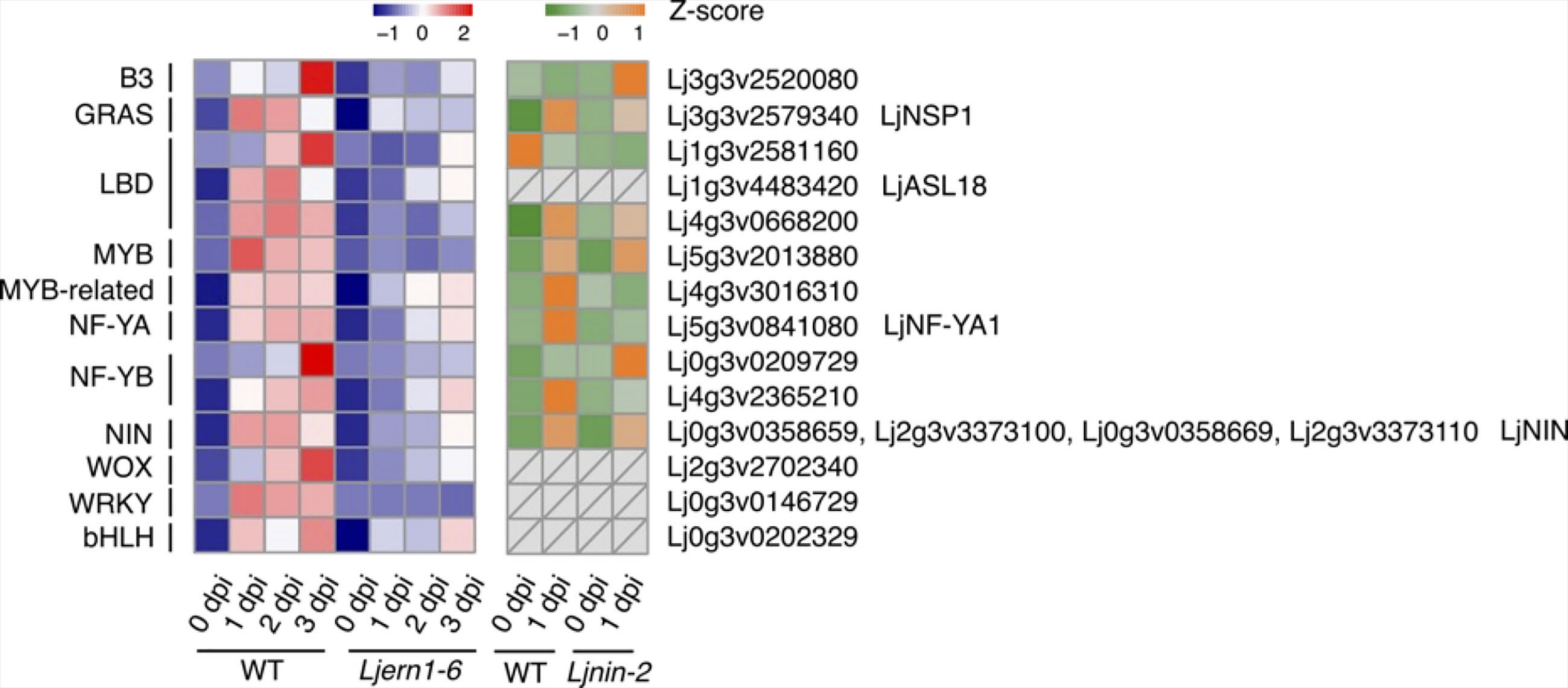
Transcript response of transcription factor genes in WT and mutants Heatmap showing expression patterns of differentially expressed transcription factor genes in WT (MG-20) and *Ljern1-6* mutants (left grid) and WT (Gifu B-129) and *Ljnin-2* mutants (right grid).

To gain a better understanding of the transcription network downstream of LjERN1, we also examined the expression of transcription factor-encoding genes in WT plants and *Ljern1-6* mutants. Based on the classification of transcription factor families and gene IDs from PlantTFDB (Jin et al., 2014; Jin et al., 2015; Jin et al., 2017; Tian et al., 2020), we analyzed the expression of 14 transcription factor genes that were decreased in *Ljern1-6* mutants (**Figure 6**). The reduced expression levels of *LjNIN* and its target genes, such as *LjNF-YA1*, were consistent with our previous findings (Liu et al., 2019b). The expression of *LjNF-YA1* and other LjNIN target genes may be solely dependent on *LjNIN* or may require regulation by both LjERN1 and LjNIN. Based on a combination of our RNA-seq results and the DNA array data, it seemed that the expression of the Myb transcription factor gene *Lj5g3v2013880* was dependent on LjERN1 but not NIN. It is possible that together with a few other transcription factor genes in the Wox, WRKY, and bHLH families, these transcription factors are involved in mediating the regulation of *LjNIN* expression downstream of LjERN1.

## Conclusion

In this present study, we transcriptionally compared gene expression profiles between WT *L. japonicus* and *Ljern1-6* mutants in response to rhizobial infection. LjERN1 affected cell wall remodeling *via* expansin, pectinases, PMEs, and PMEIs to affect infection thread formation and cortical cell division. LjERN1 may also be involved in mediating signal transduction through protein kinases, including LjEpr3. During root nodule symbiosis, phytohormone signaling is finely tuned, which may also require the involvement of LjERN1. Many of the DEGs with decreased expression in *Ljern1-6* mutants have also showed decreased expression levels in *Ljnin-2* mutants, suggesting that they were also regulated by LjNIN. This supports the theory that LjERN1 and LjNIN may have a close relationship in the regulation of gene expression. However, the present study did not determine whether the multifunctional LjERN1 is involved in rhizobial infection, nodule organogenesis, or both processes. Future work is needed to resolve this concern.

## Data availability statement

RNA sequencing data are available in the SRA database in the BioProject PRJDB13938; https://ddbj.nig.ac.jp/resource/sra-submission/DRA014481.

## Author contributions

ML, TS and MK conceived and designed the analysis; ML, AO, KY and TG collected data and performed analyses; HK and TM contributed analysis tools and performed analysis; TG deposited RNA sequence data to INSCD. ML, TS and MK wrote the paper. All authors contributed to the article and approved the submitted version.
