# Supplementary material for: The effects of ERN1 on gene expression during early rhizobial infection in Lotus japonicus
Source: Front Plant Sci. 2023 Jan 17;13:995589. doi: 10.3389/fpls.2022.995589 (PMC9888413; doi:10.3389/fpls.2022.995589)
Supplement: Supplementary file 1 [file DataSheet_1.zip › Supplementary Figures.DOCX]

Supplementary figures


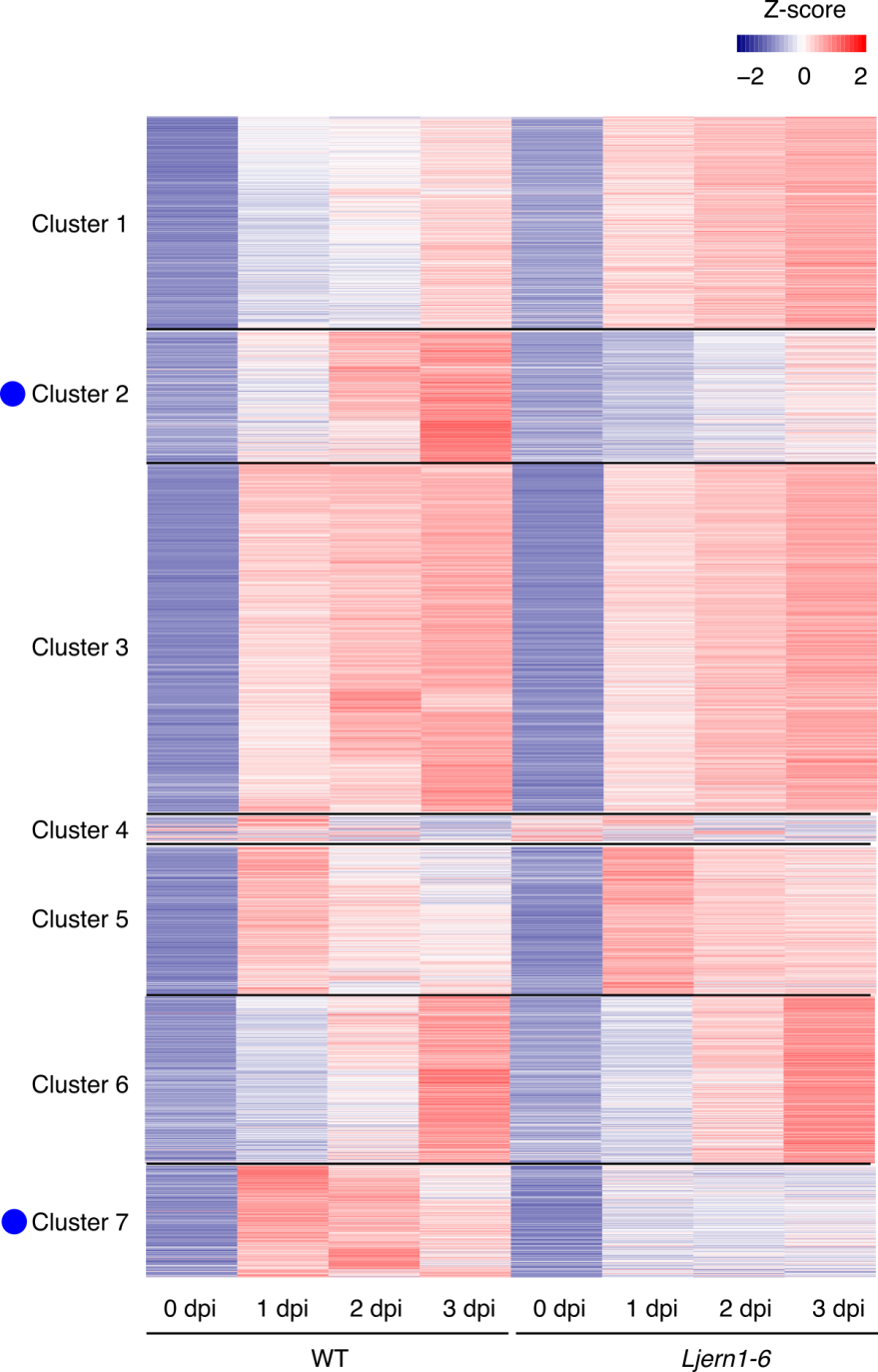


Figure S1 Clustering of 3763 infection-induced genes in WT (MG-20) using a K-means method based on their expression pattern in the WT and *Ljern1-6* mutants.

Genes in Cluster 2 and 7 showed different expression patterns between the WT and *Ljern1-6* mutants. Z-scores were used to generate a heatmap.


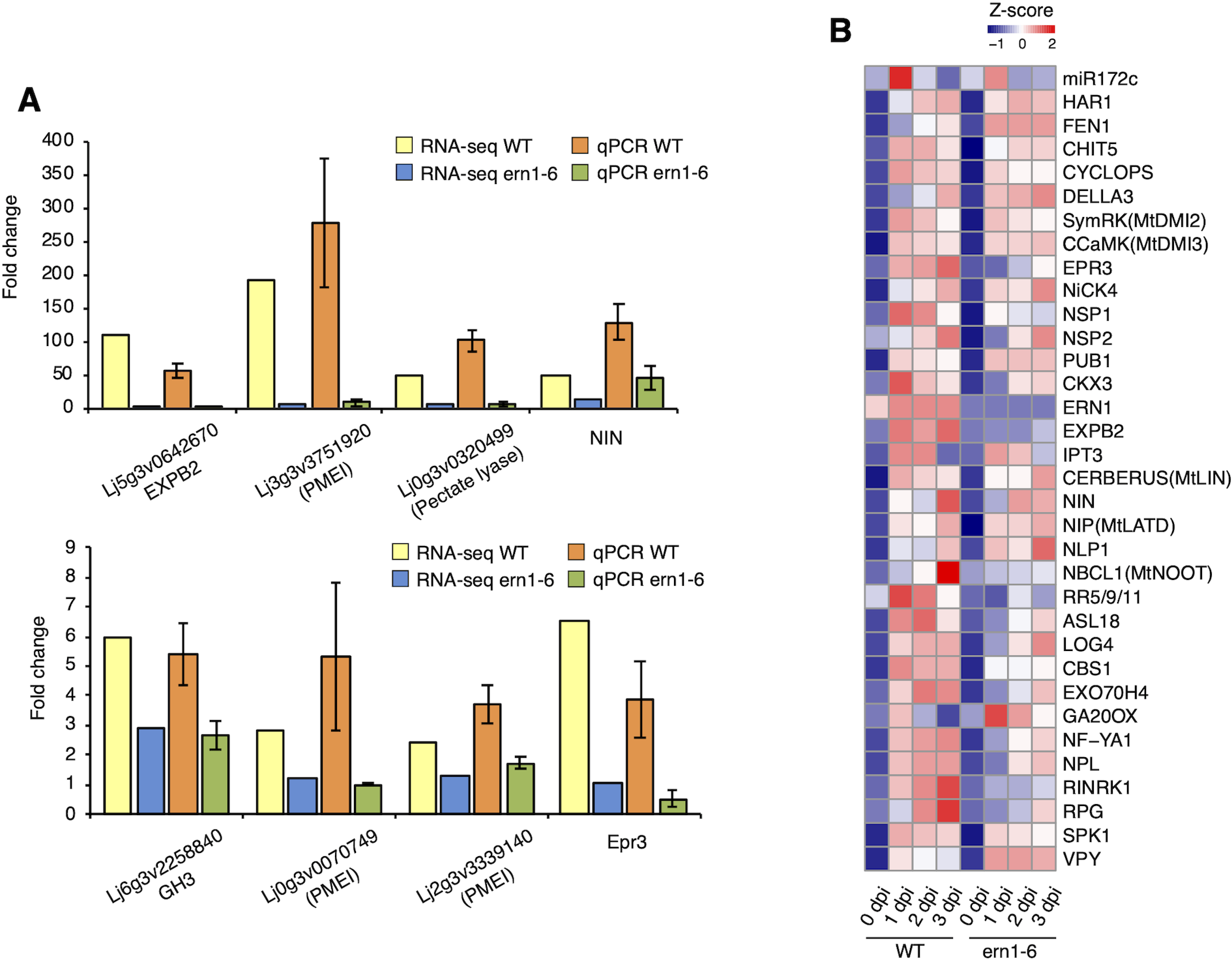


Figure S2 Validation of RNA-seq results.

(A) Expression levels of representative genes with high fold change were tested using qRT-PCR. Bar plots show the fold change in gene expression at 1 dpi compared with 0 dpi. RNA samples for qRT-PCR were the same as those used for RNA-seq library construction. (B) Expression pattern of known symbiosis genes in wild-type plants and *Ljern1-6* mutants. Genes were selected from a review by Roy et al. 2019.


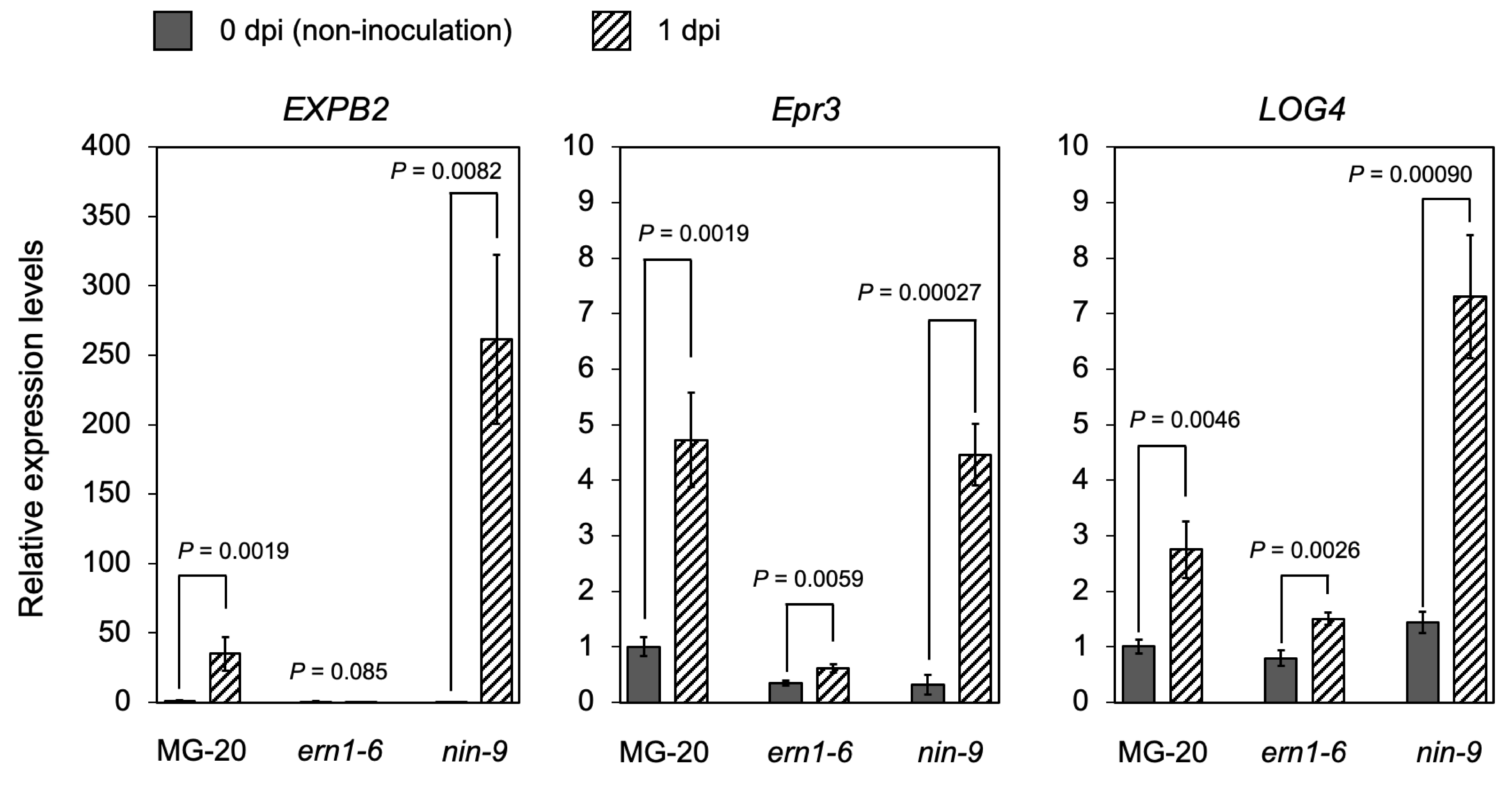


Figure S3 Genetic dependence of *EXPB2*, *Epr3*, and *LOG4* expressions at the early infection stage.

qRT-PCR analysis of these genes in MG-20 wild-type, *ern1-6*, and *nin-9* at 0 dpi (non-inoculation; gray bar) and 1 dpi (stripe bar). Data represent the average of three biological replicates with standard deviations (*n* = 10 plants for each biological replicate). Expression levels are relative to MG-20 at 0 dpi, and *LjUBQ* was used as a reference. Statistical analyses (Welch's t-test) were performed comparing 0 dpi and 1 dpi.


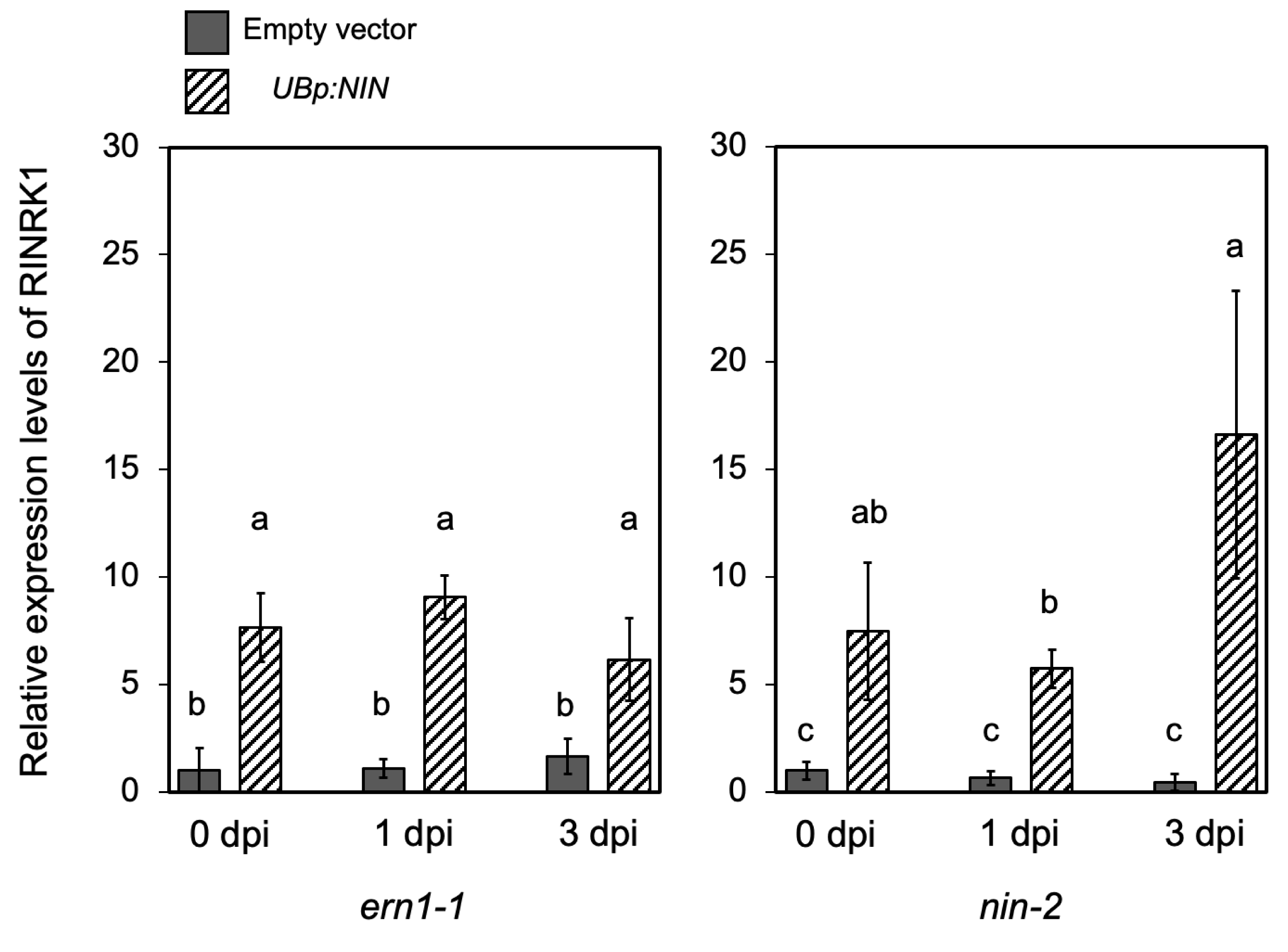


Figure S4 Effects of ectopic expression of *NIN* on *RINRK1* activation.

Transgenic hairy roots harboring empty vector (gray bar) and *UBp:NIN* (stripe bar) were generated in *ern1-1* (Left) and *nin-2* (Right). Data represent the average of three biological replicates with standard deviations (*n* = 6 to 10 plants for each biological replicate). Expression levels are relative to *ern1-1* or *nin-2* with empty vector at 0 dpi (non-inoculation), and *LjUBQ* was used as a reference. Different letters indicate Tukey’s honest significant differences (*P* < 0.05, one-way ANOVA followed by multiple comparisons).
